# Switching of both local ferroelectric and magnetic domains in multiferroic Bi_0.9_La_0.1_FeO_3_ thin film by mechanical force

**DOI:** 10.1038/srep31867

**Published:** 2016-08-22

**Authors:** Tingting Jia, Hideo Kimura, Zhenxiang Cheng, Hongyang Zhao

**Affiliations:** 1 National Institute for Materials Science, 1-2-1 Sengen, Tsukuba, Ibaraki 305-0047, Japan; 2 Institute for Superconducting & Electronic Materials, University of Wollongong, Innovation Campus, North Wollongong, NSW 2500, Australia; 3 Department of Materials Science and Engineering, Wuhan Institute of Technology, Wuhan 430073, China

## Abstract

Cross-coupling of ordering parameters in multiferroic materials by multiple external stimuli other than electric field and magnetic field is highly desirable from both practical application and fundamental study points of view. Recently, mechanical force has attracted great attention in switching of ferroic ordering parameters via electro-elastic coupling in ferroelectric materials. In this work, mechanical force induced both polarization and magnetization switching were visualized in a polycrystalline multiferroic Bi_0.9_La_0.1_FeO_3_ thin film using a scanning probe microscopy system. The piezoresponse force microscopy and magnetic force microscopy responses suggest that both the ferroelectric domains and the magnetic domains in Bi_0.9_La_0.1_FeO_3_ film could be switched by mechanical force as well as by electric field. High tip stress applied on our thin film is demonstrated as able to induce ferroelastic switching and thus induce both ferroelectric dipole and magnetic spin flipping, as a consequence of electro-elastic coupling and magneto-electric coupling. The demonstration of mechanical force control of both the ferroelectric and the magnetic domains at room temperature provides a new freedom for manipulation of multiferroics and could result in devices with novel functionalities.

Multiferroic materials combine two or three types of ferroic ordering simultaneously, such as ferroelectric, ferromagnetic, and ferroelastic orderings, and therefore, they present tremendous opportunities to investigate the coupling between these properties, which enables the dynamic manipulation of one ordering parameter by another for a broad range of potential applications in sensing, actuation, memory, etc^1^,^2^. Numerous efforts have been made to investigate the coupling phenomena (Fig. 1), especially the coupling between electric and magnetic orderings that would account for the magneto-electric (ME) effect in multiferroic materials^3^. BiFeO_3_ (BFO) has been one of the most popular and well-studied materials in the multiferroic research field, not only because of its simultaneous ferroelectricity and antiferromagnetism at room temperature, but also due to the intrinsic ME coupling at room temperature^4^,^5^,^6^. Theoretical and experimental investigations on the ME coupling in BiFeO_3_ based materials are growing rapidly, the domain switching mechanism is one of the key research issues on ME effect^7^,^8^. Meanwhile, scanning probe microscopy (SPM) based techniques have emerged as powerful characterization methods to study the domain dynamics and switching phenomenon. A spectacular example is piezoresponse force microscopy (PFM), which has opened a pathway for not only probing the polarization and electromechanical response on the nanoscale level, but also applying mechanical force locally on the material. One experimental approach to load mechanical force in nanosized ferroelectric materials is using a PFM tip onto the surface of BaTiO_3_ thin film^9^. It was argued that the flexoelectricity which is related to a tip strain gradient switches the spontaneous polarization. However, the flexoelectric effect is not a dominant factor when considering ferroelastic strain in multiaxial multiferroics by applying an external mechanical force. In application of multiferroics as non-volatile magnetoelectric devices, it is important to control the domain switching path, since ferroelasticity turns out to be coupling media between magnetic and electric orders in BiFeO_3_-based thin films^10^. Therefore, using mechanical force directly switch both ferroelectric domain and magnetic domain provide a possibility in locally mechanical manipulation of ferroelectric and magnetic orders. Although the mechanical-force-induced ferroelectric phase transition in multiferroic BiFeO_3_ thin film has been studied experimentally^11^, to the best of our knowledge, mechanical force induced switching of both magnetic and ferroelectric domains has never been realized in any multiferroic material. It has been reported that giant magneto-elastic coupling can lead to atomic displacements and thus give rise to strong ME coupling in rare earth magnets^12^, which may lay a solid foundation for the possible experimental switching of magnetic domains by mechanical force in multiferroic materials.

In order to investigate the effect of tip stress on both ferroelectric domain and magnetic domain in a multiferroic material, we use PFM to apply nanoscale mechanical force on nanomaterials through a probe tip, and scan the ferroelectric response and magnetic phase in a Bi_0.9_La_0.1_FeO_3_ thin film by changing the working mode from PFM to magnetic force microscopy (MFM). Ferroelectric domain and magnetic domain are switched by applying electric field. The mechancial force would generate both ferroelectric domain switching and ferroelastic domain switching. The ferroelastic domain switching was observed from the polarization reversal due to the combination of tip strain and elastic strain gradient in a ferroelectric thin film.

In this work, we deposited Bi_0.9_La_0.1_FeO_3_ (BLFO) thin films on Pt/TiO_2_/SiO_2_/Si substrates by pulsed laser deposition (PLD). We have visualized the switching of magnetic domains in the as-deposited BLFO thin film by applying mechanical force on the probe tip using a Nanocute SPM system. Meanwhile, we switched magnetic domains as well as ferroelectric domains using an electrical field. Therefore both ferroelectric and magnetic domain switching were successfully demonstrated by mechanical force as well as by electric field, revealing the complex coupling among polarization, magnetization, and strain in multiferroic BLFO thin film. This study provides an understanding of the ME coupling in a polycrystalline multiferroic Bi_0.9_La_0.1_FeO_3_ thin film to manipulate both ferroelectric and magnetic domains by applying mechanical force and electric field, which opens a new route to design magnetoelectric devices.

## Results and Discussion

### Structural characterization

Crystal structure characterization and component analysis of the Bi_0.9_La_0.1_FeO_3_ film on the Pt/TiO_2_/SiO_2_/Si substrate, which is an optimized composition of BiFeO_3_ with better electric performance in terms of reduction in current leakage, for our tests of mechanical switching of both ferroelectric and magnetic domains, are shown in Fig. 2. The film was found to be polycrystalline and based on a hexagonal symmetry with a space group R3c (161), with lattice parameters of *a* = 0.5746 nm, *b* = 0.5746 nm, and *c* = 1.3410 nm, and a random growth habit with a mixture of (012), (110), (202), and (024) diffraction peaks. The SEM image in the inset of Fig. 2(a) shows a cross-sectional view of the as-deposited BLFO film with film thickness of ~219 nm. The energy dispersive X-ray spectroscopy (EDX) element analysis shown in Fig. 2(b) quantitatively indicates that the amount of elements in the as-deposited BLFO thin film matches well with the target. The element mapping over the entire film shows a uniform distribution of the composition elements [Fig. 2(c)].

### Macroscale ferroelectric and magnetic properties

A large remanent polarization (2*P*_r_) of 170.7 μC/cm^2^ and a coercive field (*E*_c_) of about 303.6 kV/cm at a maximum applied field of 500 kV/cm were observed in Fig. 3(a). The ferroelectric hysteresis (*P*-*E*) loops measured at different applied fields are shown in upper inset of Fig. 3(a). Lower inset of Fig. 3(a) shows leakage current-electric field curves (*J*-*E*) during *P*-*E* loop measurements. Obviously, there are current density peaks corresponding to the switching of polarization by an external field. In addition, a linear current contribution can be identified, which suggests that the polarization of BLFO in the *P*-*E* loops contains some contribution from the leakage current. To exclude the contribution of the leakage current to the polarization and to identify the intrinsic polarization value of our film, we carried out so-called positive-up-negative-down (PUND) measurements. Figure 3(b) shows the switching polarizatiovn as a function of the applied voltage, and the inset of Fig. 3(b) shows the applied voltage waveform. The switched polarization is saturated with the switching time and consistent with the 2*P*_r_ value obtained from the hysteresis measurement. The pulsed remanent polarization value Δ*P*_r_ [switched polarization (*P*_sw_) - non-switched polarization (*P*_nsw_)] (82.7 μC/cm^2^) is approximately 1.2 times that of *P*_r_ (70.6 μC/cm^2^). Thus, the presence of intrinsic ferroelectricity in our BLFO thin film can be concluded. The magnetic characteristics of BLFO thin film was investigated as a function of temperature and magnetic field. The diamagnetic background due to the Si substrate has been subtracted from all the data. Figure 3(c) shows the in-plane magnetic hysteresis (M-H) loops measured at 300 K, the saturation magnetization is 40.5 emu/cm^3^. Field cooling (FC) and zero field cooling (ZFC) magnetization versus temperature (M-T) is presented in Fig. 3(d). The BLFO thin film exhibits typical characteristic features of a canted antiferromagnetic phase and is consistent with the character for BiFeO_3_ thin film deposited on a diamagnetic Pt/TiO_2_/SiO_2_/Si substrate.

### Manipulation of both ferroelectric and magnetic domains by electric field

Figure 4 presents the ferroelectric and magnetic characterization of the as-deposited BLFO thin film by PFM and magnetic force microscopy (MFM). We firstly poled the thin film with +15 V in a large square box with areas of 6 × 6 μm^2^, then we poled a 4 × 4 μm^2^ aera in the centre of the square by aplying a −15 V biasand finally poled a smaller 2 × 2 μm^2^ aera in the center of poled square. Figure 4(a) shows the topographic image after electrical poling, indicating that the surface morphology is not affected by poling. A clear box-in-box pattern measured in VPFM mode in an area of 10 × 10 μm^2^ is observed [Fig. 4(b)], which shows the out-of-plane (OP) ferroelectric response, where the dark and bright contrast represents upward and downward ferroelectric domains switched by electric field. Figure 4(c) is the corresponding lateral piezoresponse force microscope (LPFM) image, indicating the in-plane (IP) component, which can be inferred from the shear forces experienced by the tip. The LPFM image is very weak and inhomegenous due to the existing of some nonswitched or back switched domains in the pattern. Subsequently, we scanned the same electrically poled area of 10 × 10 μm^2^ in MFM mode using a magnetic tip without applying dc bias, and an image corresponding well to the VPFM box-in-box pattern was observed [Fig. 4(d)], indicating that the magnetic domains in the as-deposited BLFO thin film were simultaneously switched by electric field^13^. Some dark particals are observed in the MFM image, which is regarded to be caused by dust on the film surface rather than magnetic particals. The switched pattern turns out an ensemble effect of polydomain due to the polycrystalline structure of BLFO thin film. Therefore the vertical polarization is not homogeneously distributed across the poled area and some incompletely switched domains are observed, so as the magnetic domains. In Fig. 4(d), the magnetic switching pattern looks more uniform than the ferroelectric domain in Fig. 4(b), which might be attribute to the smaller size of magnetic domain. The observed ferroelectric domain reversal by applying dc voltage demonstrates the successful electrical field manipulation of ferroelectricity in our thin film. Moreover, when we applied positive dc bias in the inner box where has been switched two times in an order of +15 V and −15 V, the magnetic domains in this polycrystalline thin film could be switched back again, indicating that magnetization reversal could also be achieved by applying electric field.

The ferroelectric domain switching phenomena was studied by PFM in a contact mode in this work. The locally applied electric field induces a polarization switching and ferroelastic domain switching due to the electro-elastic coupling^10^. In a BiFeO_3_ crystal with space group R3c, the Fe magnetic moments are coupled ferromagnetically within the pseudocubic (111) planes and antiferromagnetically between adjacent planes^13^. As illustrated in Fig. 4(g), three types of switching are possible in BLFO: 71°, 109°, and 180°, classified by the angle of the rotation across the domain wall^14^ The 71° and 109° switchings are ferroelastic switching, which are related to the reorientation of antiferromagnetic plane when apply electric field along [001] axis^15^,^16^. While the 180° switching is the ferroelectric polarization switching, which may occur through the combination of 71° and 109° ferroelastic rotations. When an electric field is applied on the thin film, direct 180° polarization switching is difficult to happen due to the requirement of high activation energy, which is uausally through the combination of 71° and 109° ferroelastic switching. The antiferromagnetic ordering in BLFO is G-type, and the orientation of the antiferromagnetic magnetization is coupled to the ferroelastic strain and is always perpendicular to the ferroelectric polarization^13^. Therefore, the 71° and 109° ferroelastic switching are related to the reorientation of antiferromagnetic plane and changing the orientation of the easy magnetization plane. Thus, the electric field can change ferroelastic strain state in a nanoregion of BLFO thin film, lead to a reorientation of the antiferromagnetic order and resulting in magnetic domain switching.

To further investigate the effects of electric field on the polarization and magnetization, we observed the domain switching evolution upon increasing the applied voltage on the sample. The PFM response difference (∆*mV*) of the *Acos* image between the positive voltage switched area and the negetive voltage switched area as a function of the dc voltage is shown in Fig. 4(e). The polarization can be switched only when the applied voltage is above 12 V. It is easy to understand that no ferroelectric domain is switched when the electric field is below coercive field. Figure 4(f) shows the magnetic phase deference (∆°) as increasing dc voltage, no switching is observed at an electric field below 10 V, while a mesa stage of magnetic domain switching is observed when applied electric bias is in the range of 10–14 V, indicating a magneto-elastic coupling start to work in this voltage range. Both piezoelectric response difference and magnetic phase deference reach maximum above 18 V, indicating both ferroelectric domain and magnetic domains are fully switched. Note that the PFM scans are in contact mode, while the MFM is in lift mode, and thus, the response signals are completely different from each other. A schematic representation of PFM and MFM is presented in Fig. 4(h). During the MFM measurements, because the short-range interaction is negligible in the scanning distance, there are three interactions possible between the magnetic tip and the sample: the long-range electrostatic force (*F*_*e*_), the medium range van der Waals interactions (*F*_*vdW*_), and the magnetic force (*F*_*m*_), so the total force (*F*_*t*_) could be written as^17^: *F*_*t*_ = *F*_*vdW*_ + *F*_*e*_ + *F*_*m*_. Assuming that the *F*_*vdW*_ interaction dominates at distances below 1 nm, at the tip-sample distance of 10 nm during MFM imaging in the present work, the *F*_*vdW*_ can be neglected. The *F*_*e*_ could be compensated by applying the correct compensation voltage^17^. So, only the magnetic interaction is recorded in the trace mode of the second scan following the topographical imaging in the first scan [Fig. 4(h)].

### Switching both of ferroelectric and magnetic domains by mechanical force

Figure 5 presents a further investigation on the ferroelectric and magnetic domain switching of the as-deposited BLFO thin film by our SPM. We poled the thin film by +15 V, −15 V and +15 V dc voltage in areas of 8 × 8 μm^2^, 6 × 6 μm^2^, and 4 × 4 μm^2^ successively, and then applied a mechanical force of 600 nN without applying any dc bias in a smaller 2 × 2 μm^2^ square in the centre. Figure 5(a) is the topographic image after electric and mechanical switching, which shows that no obvious change observed on the surface. Figure 5(b) shows a VPFM image of the box-in-box lithography pattern switched by electric field and mechanical force. The −15 V switched box is brighter than 600 nN switched area. The LPFM image as shown in Fig. 5(c) is very weak, expecially no obvious contrast observed between the inner +15 V switched box and the 600 nN switched area. Figure 5(d) presents the corresponding magnetic domain switching image. The dark contrast in the 600 nN switched area in the centre indicates that a certain tip stress can switch magnetic domains in as-deposite BLFO thin film as well as an electric field.

Upon applying mechanical force directly onto the electrically poled domians, the tip stress affects the original elastic strain state in the thin film, and promotes the strain relaxation in the film. Therefore, the ferroelastic switching is improved by the applied mechanical force. It has been shown that 180° ferroelectric domain has very high energy which switching is through cooperation of 71° and 109° ferroelastic switching. Mechanical force will cause some high energy domains (180°) and incompletely switched domains (71° and 109°) pre-switched by electric field relax back to their low energy state, although it is difficult to know the domains switching route dependant on the mechanical force direction due to inhomogeneous distributions of mechanical force in the film. Therefore, the VPFM image upon application of mechanical force has less contrast in comparison to the electric field switched pattern. As shown in Fig. 5(b), the mechanical force switched region in the center is not as bright as the outer box switched by −15 V, indicating an incomplete polarization switching. A reorientation of the antiferromagnetic orders happens when the elastic strain relaxes, due to magnetoelastic coupling, and results in magnetic domain switching as shown in Fig. 5(d).

In addition, the tip stress can also generate electric polarization (*P*_i_) in thin film through the following equation^18^:


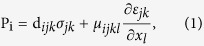


The first term is the dielectric response, where *d*_*ijk*_ is the piezoelectric coefficient, and *σ*_*jk*_ represents the stress uniformly distributed across the film; the second term refers to the flexoelectric response, where *μ*_*ijkl*_ is the flexoelectric coefficient, and 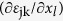 is the strain gradient. The piezoelectric effect related homogeneous strain which is linear in respect to the applied field (within the elastic limitation) could help reduce the energy barrier for domain switching, but itself could not switch the domains. Flexoelectric effect could also have possibility to switch polarization^19^. However, the order of magnitude of polarization from flexoelectric effect is much lower than that from ferroelastic strain.

To further investigate the effects of mechanical force on the polarization and magnetization, we observed the domain switching while increasing the loading force on the sample. The PFM response difference (∆*mV*) of the *Acos* image between the electrically switched area and the force switched area as a function of the loading force is shown in Fig. 5(e). Initially, the switching polarization difference increases as the loading force increases, and then, at a loading force of 600–700 nN, the switching polarization difference reaches a maximum. With further increasing the loading force, the piezoresponse difference decreases. As the mechanical force increasing to 600 nN, more and more ferroelastic domains are relaxed and switched by force. When the mechanical force is higher than 600 nN, ferroelastic domains with antiparallel sign start to be switched. Thus, the polarization in the thin film allowed for ferroelastic rotation reach a maximum and then decreased due to the change of polarization from the previously poled area by +15 V. Figure 5(f) presents a similar trend as Fig. 5(e) in the magnetic phase reversal (∆°), interpreted by magnetic phase response in MFM images as a function of loading force, which shows that the magnetic switching reaches a maximum at 600 nN, indicating an inherent relationship between the magnetic domain switching and the ferroelastic domain switching in the BLFO thin film by mechanical force.

We realized that the mechanical force induced ferroelectric and magnetic domain switching can only be observed when these domains were initially switched by electric field. Applying a mechanical force directly on the virgin surface of the as-deposited thin film seems to have little effect on both ferroelectric and magnetic domains. It is possible that when the mechanical force was applied on the fresh polycrystalline film with randomly arranged domains, although a strain was generated which would switch individual domains, no integrate domain switching could be observed due to the polydomain nature of polycrystalline thin film. Thus, there is still no obvious contrast observed in both PFM and MFM image due to the random distribution of ferroelectric domains and magnetic domains after mechanical switching without an initial electrical switching. As shown in supplementary Figure S1, after applying mechanical force the switched ferroelectric domains and magnetic domains could be reversed again by applying electric field, indicating a cross-coupling in ferroelectric, antiferromagnetic, and ferroelastic orders in BLFO thin film.

Full analysis of the mechanical stress and electric field distribution in our system is outside the scope of this work and will be discussed in a future article. Lu *et.al* has reported the flexoelectricity induced by the strain gradient from tip stress result in ferroelectric domain switching in an epitaxy BaTiO_3_ thin film, in which the piezoelectric effect only generates homogeneous stress and remains unaffected to the polarization reversal^20^. In a multiaxial ferroelectrics such as BLFO with a rhombohedral structure, flexoelectricity is not a dominant factor compared with the ferroelasiticity during domain switching^10^. A large enough external strain should lead to reorientation of electrically poled ferroelectric/ferroelastic domains. Due to the fact that our BLFO film is polycrystalline with non-epitaxial growth, the observed switching behavior by PFM is a collective behavior of all the switchable dipoles under the electric field perpendicular to the surface, and the VPFM and LPFM images result from the projections of these dipoles along different directions. The true orientation of the polarization can be deconvoluted only if all the components of the pizoelectric tensor are known^21^. Because the orientations of the individual grains are random for our polycrystalline BLFO thin film, quantitative analysis of the switching of these domains is difficult.

### Exclusion of screening charge effect

In the process of scanning the film surface with a PFM tip, charge injected into the film from the charged tip will accumulate at the film surface. In addition, there is also screening charge accumulated at the polar surface. All these charges at the film surface will give a false response in the PFM phase image and cover/mask the intrinsic polarization response. Therefore, we carried out further work to eliminate the screening charge effect. A VPFM lithographic image is shown in Fig. 6(a), and the switching process is the same as that mentioned above. Next, the scanning tip and the bottom electrode were short-circuited, and no contrast was observed during the short-circuit process, as shown in Fig. 6(b), with reference to the screening of the film surface charges; finally, we read the area again without the short-circuit between the top and bottom electrodes, and found that the lithographic pattern could still be obtained [Fig. 6(c)]. The pattern, which showed strong contrast caused by the polarity of the surface after applying the electric fields and loading forces, was almost the same as the pattern scanned before the short-circuit, indicating that the observed phase contrast is intrinsically from the switching of ferroelectric domains^22^. Figure 6(d) presents a schematic diagram of the measurement.

We note that these promising results notwithstanding, our observations are still early in our understanding of the details of the cross-coupling in such polycrystalline thin film. The exact details of factors such as crystal grain boundaries, domain walls movement, the switching time during the ferroelectric and magnetic domain switching, the coupling energy between the ferroic orders in the multiferroic thin films etc. should be carefully addressed in future experimental and fundamental investigation.

## Conclusion

In summary, we have demonstrated that both ferroelectric and magnetic domains in our Bi_0.9_La_0.1_FeO_3_ thin film on Pt/TiO_2_/SiO_2_/Si substrate could be switched by loading mechanical force and applying electric field. We have proved that the external mechanical force introduce a strain effect on the ferroelastic state in the thin film, which is a coupling path of the magnetolelectric effect. An external mechanical force can emerge as a practical means to control both ferroelectric and magnetic domains in BLFO thin film. This result provides a pathway towards understanding the ferroelectric-magnetic-elastic coupling mechanism in multiferroic materials. Furthermore, it will enrich our understanding of the tip stress induced effects, which will open up new technical opportunities for multifunctional device designs, such as high density data storage via mechanical means, nanoscale sensors, detectors, etc.

## Methods

The crystal structure was evaluated by X-ray diffraction (XRD) using a diffractometer (RIGAKU RINT 2000). The film thickness was measured using a JSM-6500F scanning electron microscope (SEM). For electrical measurements, a Pt top electrode was coated on the surface of the BLFO thin film through a shadow mask with a diameter of 100 μm to form a capacitor. The ferroelectric properties were measured at room temperature (RT) with an aixACCT TF-1000 ferroelectric tester.

A NanoCute SPI 3800 (Hitachi HiTech Science) SPM system which enables both piezoresponse force microscopy (PFM) and magnetic force microscopy (MFM) measurements was used to investigate local piezoelectric/ferroelectric and magnetic properties at room temperature. A conduction tip (Si cantilever coated with Rh) was used to apply electric field and mechanical force in PFM measurements, while a magnetic tip coated with CoPtCr film was used in the MFM measurements. The reason for not using the MFM tip to do force scanning was to avoid the magnetization effect from the very weak magnetic field of the MFM tip. The mechanical constant was 15 N/m, and the resonance frequency was 139 kHz. A schematic representation of both PFM and MFM is presented in Fig. 4(f).

The PFM mode is driven electrically using contact mode atomic force microscope (AFM) feedback for topographic tracing, and a periodic bias *V*_*tip*_ = *V*_*dc*_ + *V*_*ac*_cos(*ωt*) is applied on the tip. The tip deflection is *A* = *A*_*0*_ + *A*_*1ω*_cos(*ωt* + *θ*), where the deflection amplitude *A*_1ω_ is determined by the tip motion, and the phase (*θ*) indicates the orientation of the atomic polarization^23^. When the electric field is applied to the piezoelectric material, domains with an upward polarization vector contract with a negative voltage, producing a phase shift of *θ* = 0°. For the downward domains, the situation is reversed, and *θ* = 180°. The piezoresponse amplitude (*A* = *A*_*1ω*_* /V*_*ac*_) defines the local electromechanical activity of the surface^24^. In our Nanocute SPM system, the PFM image is recorded as an *Acos* image which combines both amplitude and phase components, making it easier to observe both the polarization and the electromechanical response in the sample using our SPM. In this work, *Acos* images were recorded with topographic images, applying a signal at 5 kHz with a 2 V oscillation under a ±15 V dc bias. We didn’t apply dc bias on the tip when using mechanical force to switch the sample. The force is applied only in VPFM mode. The scan rate was 5 kHz, corresponding to a tip-surface contact time of 500 s/nm, so the scan is comparable to locally applying a force, and the mechanical force indicated by the PFM tip can be effectively applied on the sample.

Each MFM scan included two steps: firstly, the trace was recorded in tapping mode to image the topography of the surface; and the second step was in lift mode, in which the tip does not come into contact with the film surface, to assess the stray magnetic field perpendicular to the surface. The lift distance of the probe tip was about 10 nm during the MFM measurements.

In order to plot the PFM polarization switching as a function of loading force and dc voltage [Fig. 4(e,g)], we measured the polarization switching by recording the response voltage difference (∆*mV*) between the positive voltage switched area and the mechanically switched area or negative voltage switched area in the *Acos* image [inset of Fig. 4(e,g)]. The corresponding magnetic switching as a function of loading force was also recorded in terms of the magnetic difference (∆°) in the MFM phase images [inset of Fig. 4(f,h)].

## Additional Information

**How to cite this article**: Jia, T. *et al.* Switching of both local ferroelectric and magnetic domains in multiferroic Bi_0.9_La_0.1_FeO_3_ thin film by mechanical force. *Sci. Rep.* **6**, 31867; doi: 10.1038/srep31867 (2016).

## Supplementary Material

Supplementary Information

## Figures and Tables

**Figure 1:**
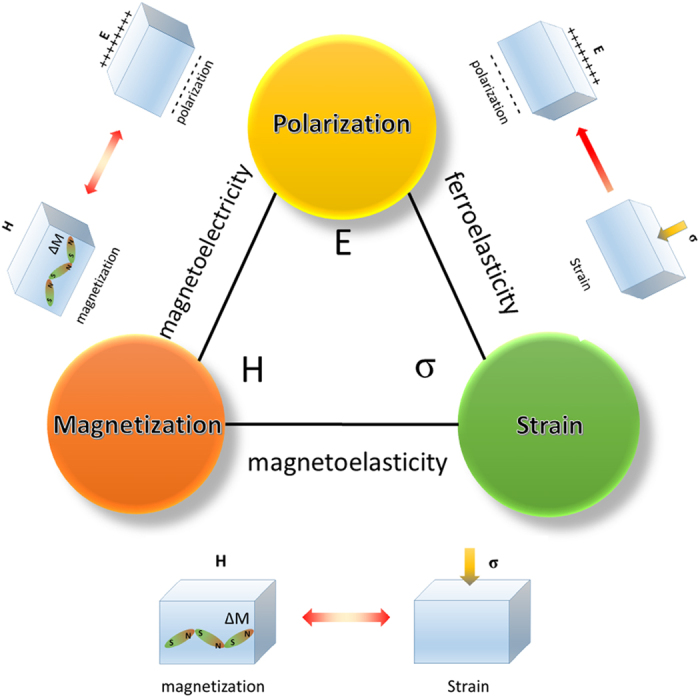


**Figure 2:**
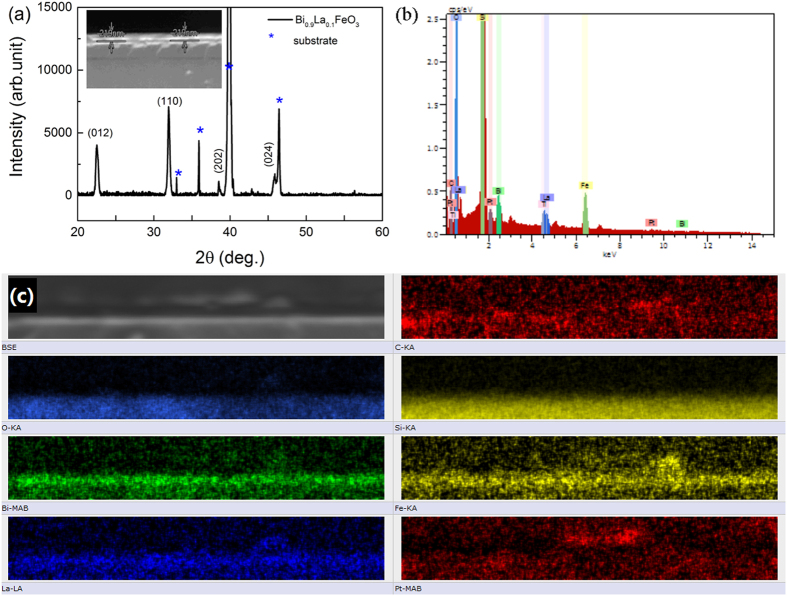
(**a**) XRD pattern of Bi_0.9_La_0.1_FeO_3_ thin film deposited on Pt/TiO_2_/SiO_2_/Si substrate; the inset showing a cross-sectional SEM image of the as-deposited thin film; film thickness of as-deposited thin film is ~219 nm, at this thickness, a piezoresponse can be recorded easily, and the minimum switching field increases when the film thickness is increased. (**b**) EDX element analysis spectrum, and (**c**) element mapping.

**Figure 3:**
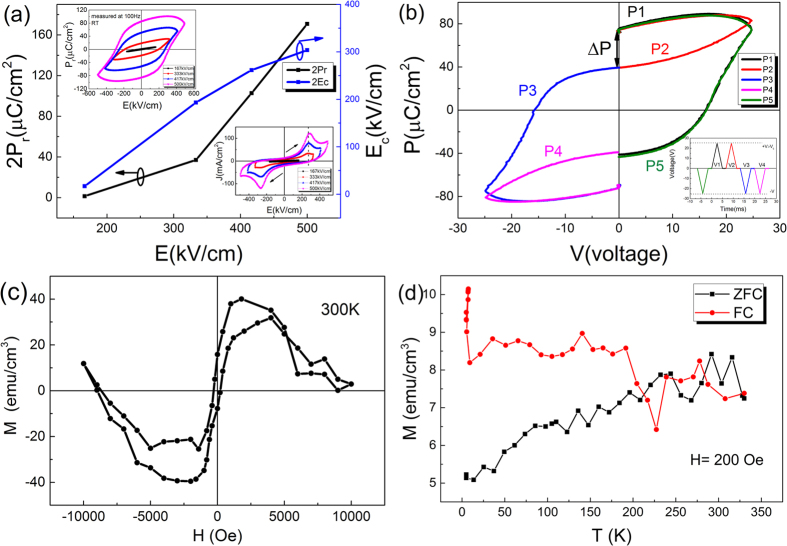
(**a**) Ferroelectric measurements of Pt/BLFO/Pt capacitor: remnant polarization and coercive field as a function of electric field; upper inset shows ferroelectric polarization - electrical field (P-E) hysteresis loops measured at different electric fields; lower inset shows leakage current - electric field curves during P-E loop measurements; (**b**) Positive-up negative-down (PUND) switching polarization as a function of voltage; inset is waveform of the applied triangle pulse with a 1 ms read pulse time and 1 ms delay. (**c**) Magnetic hysteresis loop (M-H) of Bi_0.9_La_0.1_FeO_3_ (BLFO) thin film measured at 300 K. (d) Magnetization-temperature (M-T) dependence of field cooling - zero field cooling (FC-ZFC) curves for the BLFO thin film with application of a magnetic field of 200 Oe.

**Figure 4:**
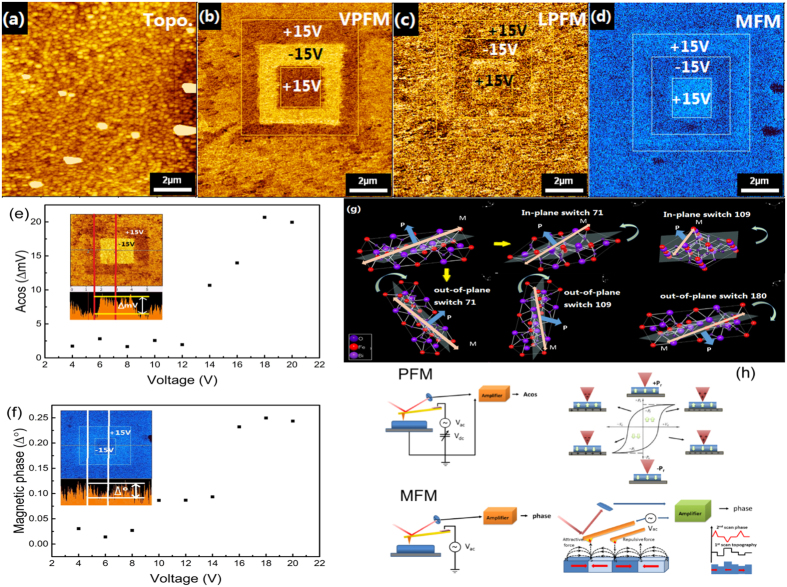
Electric control of ferroelectric domain and magnetic domain in a polycrystalline BLFO thin film. (**a**) Topographic image, (**b**) VPFM, (**c**) LPFM and (**d**) MFM images after electric poling. (**e**) and (**f**) are PFM response difference (∆*mV*) and MFM phase difference (*∆*°) area as a function of applied voltage between the positive and negtive electrical switched region, respectively; (**g**) Schematic illustrations of the three kinds of domains permitted in BiFeO_3_ for in-plane and out-of-plane orientations. (**h**) Schematic illustrations of PFM and MFM operations.

**Figure 5:**
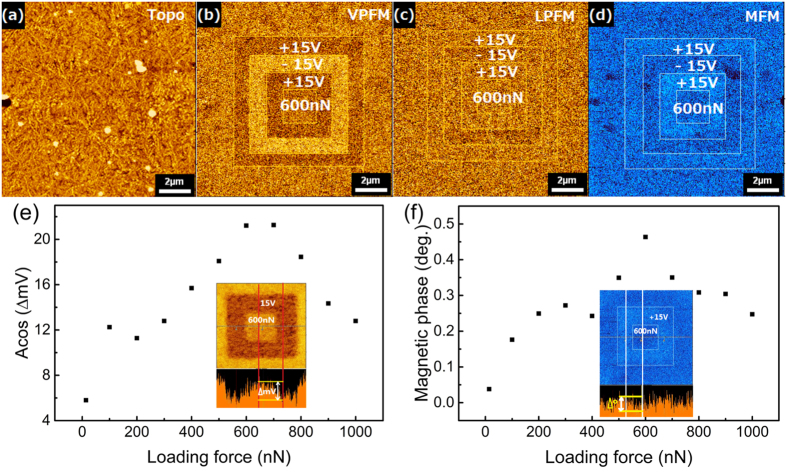
Domain switching in Bi_0.9_La_0.1_FeO_3_ thin film controled by dc voltage and mechanical force using SPM: (**a**) Topographic image, the surface roughness is measured by a root mean square (RMS) value of 3.5 nm in an area of 10 × 10 μm^2^; (**b**) VPFM and (**c**) LPFM box-in-box lithographic images poled by alternate dc voltage of ±15 V and 600 nN, and (**d**) corresponding MFM image scanned in the same area. (**d**) PFM response difference (∆*mV*) of *Acos* image between the electrically switched area and the mechanical force switched area as a function of the loading force obtained in the Nanocute SPM system, (**e**) MFM phase difference (*∆*°) between the electrically switched area and the mechanically switched area. Insets of (**d**) and (**e**) illustrate the collection of PFM response difference and magnetic phase difference measurements, respectively.

**Figure 6:**
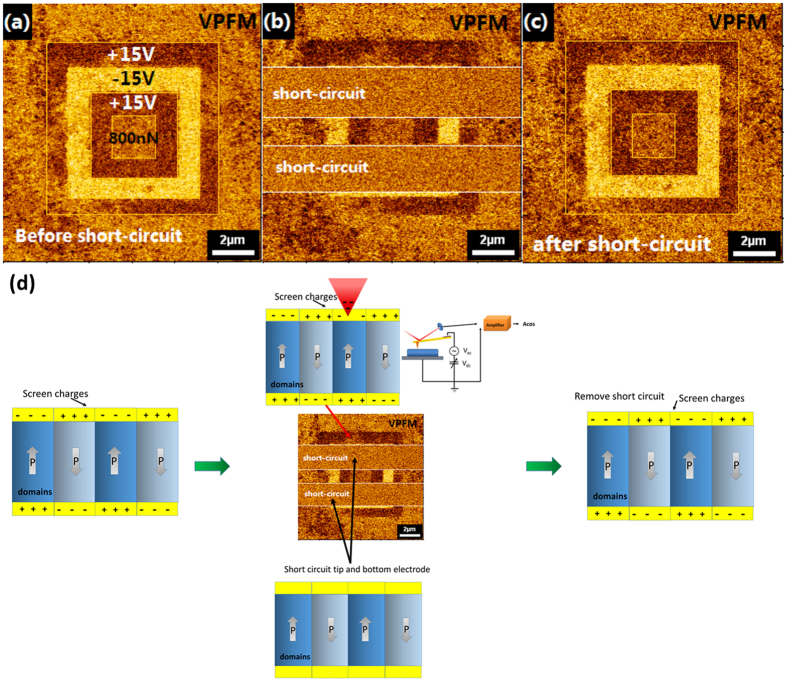
Vertical piezoresponse force microscope (VPFM) images of Bi_0.9_La_0.1_FeO_3_ thin film: (**a**) Lithographic pattern created by alternate dc bias of ±15 V and external mechanical force of 800 nN; (**b**) short-circuiting of the bottom electrode and the cantilever during pattern reading; (**c**) PFM image scanned after removal of the short-circuit. (**d**) Schematic diagram of the measurement patterns corresponding to the polarization directions.
