# Supplementary material for: Switching of both local ferroelectric and magnetic domains in multiferroic Bi0.9La0.1FeO3 thin film by mechanical force
Source: Sci Rep. 2016 Aug 22;6:31867. doi: 10.1038/srep31867 (PMC4992825; doi:10.1038/srep31867)
Supplement: Supplementary Information [file srep31867-s1.pdf]

Supporting Information

Switching of both local ferroelectric and magnetic domains in multiferroic $Bi_{0.9}La_{0.1}FeO_3$ thin film by mechanical force

Tingting Jia, Hideo Kimura*, Zhenxiang Cheng*, and Hongyang Zhao

Dr. T. T. Jia, Dr. H. Kimura
National Institute for Materials Science, 1-2-1 Sengen, Tsukuba, Ibaraki 305-0047, Japan
E-mail: jia.tingting@nims.go.jp and kimura.hideo@nims.go.jp
Dr. Z. X. Cheng
Institute for Superconducting & Electronic Materials, University of Wollongong, Innovation Campus, North Wollongong, NSW 2522, Australia
E-mail: cheng@uow.edu.au
Dr. H. Y. Zhao
Department of Materials Science and Engineering, Wuhan Institute of Technology, Wuhan 430074, China

Keywords: Scanning probe microscopy, bismuth ferrite, flexoelectric, mechanical force, magnetic domain.

Film deposition.

$Bi_{0.9}La_{0.1}FeO_3$ (BLFO) thin film was deposited on $Pt/TiO_2/SiO_2/Si$ substrates using a pulsed laser deposition (PLD) system with the laser source at 355 nm and a repetition rate of 10 Hz. The ceramic target for BLFO deposition was prepared using a conventional solid-state reaction process. The BLFO thin film was deposited at 550 °C over a period of 30 min, and the sample was then cooled down to room temperature (RT). The detailed deposition conditions can be found in the previous reports of our group.[1, 2]

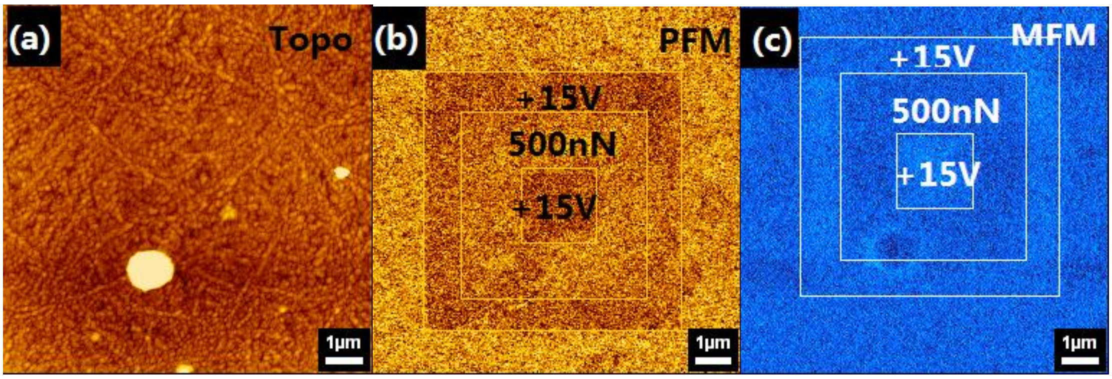

Figure S1. Electrical reversal the mechanical switched domains in BLFO thin film. (a) Topographic image, (b) VPFM image, the film is firstly switched by a positive dc voltage of +15V, then switched back by a mechanical force of 500nN, at last a positive dc voltage is applied in the center to reverse the polarization; (c) corresponding MFM image, indicating the mechanical switched magnetization could also be reverted by electric field.

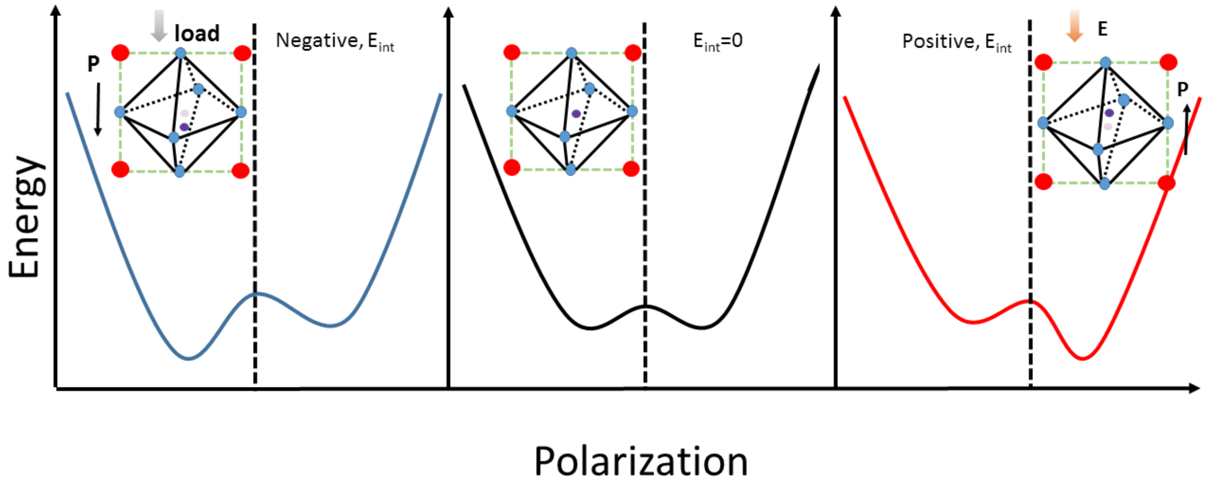

Figure S2. Schematic illustration of the relationship between free energy and polarization. The free energy of the BLFO film: (a) with mechanical force, (b) without any external mechanical force or electric field, (c) with positive electric field.

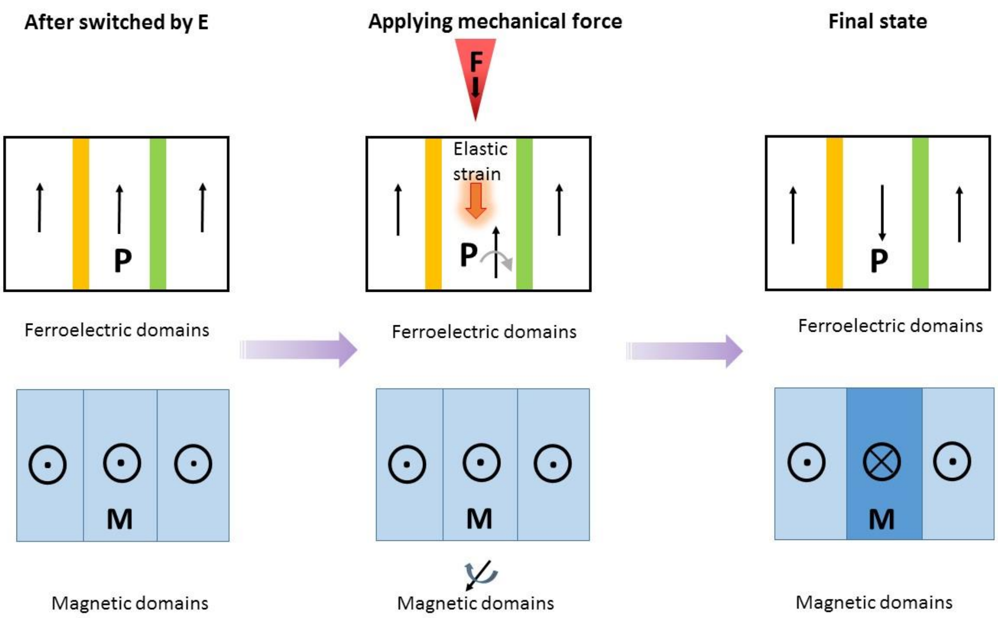

Figure S3. Illustration of ferroelectric domain switching and magnetic domain switching when applying mechanical force.

References

1. Cheng ZX, Wang XL, Kimura H, Ozawa K, Dou SX. La and Nb codoped $BiFeO_3$ multiferroic thin films on $LaNiO_3$/Si and IrO_2/Si substrates. *Appl Phys Lett* **92**, 092902 (2008).
2. Cheng ZX, Wang XL, Dou SX, Kimura H, Ozawa K. Improved ferroelectric properties in multiferroic $BiFeO_3$ thin films through La and Nb codoping. *Phys Rev B* **77**, 092101 (2008).
